# Supplementary material for: Analysis of Circulating Tumor and Cancer Stem Cells Provides New Opportunities in Diagnosis and Treatment of Small Cell Lung Cancer
Source: Int J Mol Sci. 2022 Sep 17;23(18):10853. doi: 10.3390/ijms231810853 (PMC9503899; doi:10.3390/ijms231810853)
Supplement: Supplementary file 1 [file ijms-23-10853-s001.zip › ijms-1887826-supplementary.pdf]

Table S1. Characterization of T-lymphocytes and cancer stem cell markers.

Marker	Characteristic	Reference
CD3	Surface marker of mature cytotoxic and helper T-lymphocytes.	[45,66]
CD8	Marker characteristic of cytotoxic T-lymphocytes. $CD8^{+}$ T-lymphocytes are the most powerful effectors in the anti-cancer immune response.	[66,45–47]
Ki67	A marker of cell proliferation.	[48]
CD69	A marker of early T-cell activation expressed on the surface of activated T-lymphocytes.	[49,50]
EGF	The EGF receptor is a classic receptor tyrosine kinase. EGF receptors are expressed on a plethora of cell types, including lymphocytes, epithelial, endothelial, neuronal and glial, bone, adipose, liver, and cardiovascular cells. EGF receptor overexpression has been documented in many tumors. Effector T-lymphocytes expressing EGF receptor, proliferate better and produce more IFN-γ and TNF-α in the presence of EGFR ligands produced by tumour cells *in vitro* and show a stronger antitumor response, delaying tumour growth *in vivo.*	[27]
Axl	AXL is a receptor tyrosine kinase (RTK) involved in proliferation, migration, invasion, survival, apoptosis, and tumour angiogenesis. It is expressed in normal tissues, especially in the bone marrow stroma and myeloid cells, immune cells, including dendritic cells, macrophages and NK cells, as well as in tumour cells and the tumour vasculature. Axl weakens the antitumor response by modulating the activity of immune cells.	[51–53]
CD87	Participates in cell migration, regulates cell adhesion. High CD87 expression correlates with poor clinical outcome and significantly shorter overall survival in SCLC. $CD87^{+}$ cell population demonstrated a high spherical ability, an increased tumour initiation potential, and significant resistance to traditional chemotherapeutic agents in SCLC therapy.	[5,54–58]
CD117	CD117 (c-KIT) is a type III receptor tyrosine kinase. c-KIT is activated (phosphorylated) by binding of its ligand with stem cell factor (SCF). This leads to activating of signal cascade which activation apoptosis, cell differentiation, proliferation, chemotaxis, and cell adhesion. Overexpression of SCF and CD117 is observed in lung cancer. High SCF expression in lung adenocarcinoma is associated with poor prognosis. Overexpression of CD117 in lung tumours is also associated with poor prognosis, low survival, and chemoresistance.	[54,57,59,60]
ALDH	High activity of aldehyde dehydrogenase (ALDH) has been found in stem and progenitor cells. High activity and/or overexpression of ALDH can be used as a marker of CSC in various types of cancers, including lung cancer. Overexpression of ALDH1 is associated with a poor prognosis in patients with lung cancer and more severe histological grade and stage of the disease.	[61–66]
CD44	CD44 is expressed on endothelial and mesenchymal cells, cancer stem cells. Identified as a lymphocyte receptor responsible for homing. CD44 has been recognized as a lung cancer stem cell marker.	[43]
CD276	CD276 is a type I transmembrane glycoprotein and a member of the B7 regulatory protein family. It is predominantly a co-inhibitory molecule of T-cells with a partial co-stimulatory function. CD276 is an immune checkpoint molecule in the epithelial-mesenchymal transition (EMT) pathway. CD276 plays an important role in cell proliferation, invasion, and migration in malignant neoplasms. It is important to note that CD276 is present only in pathological blood vessels and absent in physiological blood vessels.	[67]

Table S2. Characteristics of the patient G and volunteer K.

Characteristic	Patient G	Volunteer K
**Age (years)**	75	35
**Sex**	male	male
**Smoke**	smokers	non-smokers
**COPD**	with COPD	without COPD
**Lung cancer**	SCLC<br>(neuroendocrine carcinoma)<br>stage III	negative

COPD – chronic obstructive pulmonary disease
SCLC – small cell lung cancer

Table S3. Patient characteristics.

Characteristic	Number of the patients with SCLC, % (n)
**Age (years)**	
Median	56.6 ± 1.3
**Sex**	
Male	85.7% (n=6)
Female	14.3% (n=1)
**Smoke**	
smokers	42.8% (n=3)
non-smokers	28.6% (n=2)
unknown	28.6% (n=2)
**COPD**	
With COPD	57.2% (n=4)
Without COPD	42.8% (n=3)
**Histopathology**	
SCLC<br>(neuroendocrine carcinoma)	100% (n=7)
**Stage of disease (AJCC)**	
Stage II	28.6% (n=2)
Stage III	28.6% (n=2)
Stage IV	42.8% (n=3)

COPD – chronic obstructive pulmonary disease
SCLC – small cell lung cancer
